# Comparative Evaluation of Differentiation Potential of Menstrual Blood- *Versus* Bone Marrow- Derived Stem Cells into Hepatocyte-Like Cells

**DOI:** 10.1371/journal.pone.0086075

**Published:** 2014-02-05

**Authors:** Sayeh Khanjani, Manijeh Khanmohammadi, Amir-Hassan Zarnani, Mohammad-Mehdi Akhondi, Ali Ahani, Zahra Ghaempanah, Mohammad Mehdi Naderi, Saman Eghtesad, Somaieh Kazemnejad

**Affiliations:** 1 Reproductive Biotechnology Research Center, Avicenna Research Institute, ACECR, Tehran, Iran; 2 Nanobiotechnology Research Center, Avicenna Research Institute, ACECR, Tehran, Iran; 3 Immunology Research Center, Iran University of Medical Sciences, Tehran, Iran; 4 Department of Biochemistry and Molecular Biology, University of Maryland School of Medicine, Baltimore, Maryland, United States of America; Cardiological Center Monzino, Italy

## Abstract

Menstrual blood has been introduced as an easily accessible and refreshing stem cell source with no ethical consideration. Although recent works have shown that menstrual blood stem cells (MenSCs) possess multi lineage differentiation capacity, their efficiency of hepatic differentiation in comparison to other stem cell resources has not been addressed so far. The aim of this study was to investigate hepatic differentiation capacity of MenSCs compared to bone marrow-derived stem cells (BMSCs) under protocols developed by different concentrations of hepatocyte growth factor (HGF) and oncostatin M (OSM) in combination with other components in serum supplemented or serum-free culture media. Such comparison was made after assessment of immunophenotye, trans-differentiation potential, immunogenicity and tumorigeicity of these cell types. The differential expression of mature hepatocyte markers such as albumin (ALB), cytokeratin 18 (CK-18), tyrosine aminotransferase and cholesterol 7 alpha-hydroxylase activities *(CYP7A1)* at both mRNA and protein levels in differentiating MenSCs was significantly higher in upper concentration of HGF and OSM (P1) compared to lower concentration of these factors (P2). Moreover, omission of serum during differentiation process (P3) caused typical improvement in functions assigned to hepatocytes in differentiated MenSCs. While up-regulation level of *ALB* and *CYP7A1* was higher in differentiated MenSCs compared to driven BMSCs, expression level of *CK-18*, detected level of produced ALB and glycogen accumulation were lower or not significantly different. Therefore, based on the overall comparable hepatic differentiation ability of MenSCs with BMSCs, and also accessibility, refreshing nature and lack of ethical issues of MenSCs, these cells could be suggested as an apt and safe alternative to BMSCs for future stem cell therapy of chronic liver diseases.

## Introduction

Cell therapy, using human hepatocytes, is being regarded worldwide as an alternative approach to organ transplantation for liver failure. Major obstacles, including donor organ shortage have encouraged scientists and physicians to take advantage of stem cells for cell therapy of liver disorders [1]. Adult bone marrow has commonly been known as the most conventional stem cell source in the field of regenerative medicine and tissue engineering, including liver tissue engineering, because bone marrow-derived mesenchymal stem cells (BMSCs) do not present the ethical issues of embryonic stem cells (ESCs), have hepatogenic differentiation ability *in vitro* and *in vivo* [2], [3] and exhibit immunosuppressive capabilities [4], [5]. However, problems such as less availability, invasive methods for sample collection and lower proliferation capacity in comparison with ESCs limit applicability of BMSCs for clinical therapy of liver diseases. Pertaining to other sources of stem cells and regardless of great achievements in generating terminally-differentiated hepatocyte-like cells from human induced pluripotent stem (iPS) cells, limitations such as the risk of tumor formation are yet to be addressed in this stem cell type [6], [7].

Several studies have reported that menstrual blood (MB) contains a unique population of cells with properties similar to adult stem cells [8]–[11]. It has been proposed that MB contains circulating BMSCs, which contribute to endometrial regeneration [12]. Menstrual blood-derived stem cells (MenSCs) exhibit a long term self-renewal ability, greater proliferation capacity compared to BMSCs and have minimal risk of karyotypic abnormalities [8]–[11], [13]. In addition, recent studies have showed that reprogramming efficiency for generation of iPS cells could be increased using MenSCs as a cell source even in the absence of ectopic expression of c-Myc [14], [15]. These characteristics, as well as the ease of access and the possibility of cyclic sample collection, make MB an appropriate stem cell supply for tissue engineering and regenerative medicine.

We have previously presented evidence on the potential of MenSCs to generate hepatocyte-like cells [16]. In the present study, we sought to compare for the first time the hepatogenic differentiation potential of MenSCs to that of BMSCs.

To date, different concentrations of hepatocyte growth factor (HGF) and oncostatin M (OSM), as main factors involved in hepatocyte development and maturation, in combination with other cytokines and growth factors, such as epidermal growth factor (EGF), basic fibroblast growth factor (b-FGF) and insulin-like growth factor (IGF), have been used to generate both biochemically- and metabolically-active hepatocyte-like cells from BMSCs. Indeed, chemical compounds such as dexamethasone (Dexa), retinoic acid (RA), sodium butyrate, nicotinamide (NTA), norepinephrine, and dimethylsulfoxide (DMSO) are known to promote hepatic differentiation of BMSCs [17]–[20], [21]. Moreover, much effort is underway to induce hepatic differentiation of BMSCs under serum-free conditions for human trial applications [22]–[25], [26]. In this study, we have presented some data about: 1) unique immunophenotypic characteristics and trans-differentiation capability of MenSCs compared to BMSCs, and 2) chromosomal stability, and non-immunogenic and non-tumorigenic nature of MenSC. As a main goal, differentiation potential of MenSCs into hepatocyte-like cells has been compared to BMSCs under three protocols developed using combinations of HGF and OSM with other components in both serum-supplemented and serum-free culture media.

## Materials and Methods

### Isolation of MenSCs and BMSCs

Menstrual blood was collected from healthy females aged 25–35 years using a Diva cup (Diva International Co., Lunette, Finland) on day 2 of menstrual cycle. All donors were given a complete description of the study and provided written informed consent before sampling. This study was approved by the Medical Ethics Committee of Avicenna Research Institute.

The contents of Diva cup were transferred into tubes containing 2.5 µg/ml fungizone (Gibco, Scotland, UK), 100 µg/mL streptomycin, 100 U/mL penicillin (Sigma-Aldrich, MO, USA) and 0.5 mM EDTA in phosphate buffered saline (PBS) without Ca^2+^ or Mg^2+^. Isolation of stem cells from menstrual blood was performed as described previously [27], [28].

Bone marrow aspirates (5–10 mL) were obtained from iliac crests of human donors at the Bone Marrow Transplantation Center, Shariati Hospital, Tehran, Iran. Samples were harvested after getting signed informed consent. This work was approved by the Medical Ethics Committee, Ministry of Health, Iran. Bone marrow stem cells (BMSCs) were isolated using a combination of density gradient centrifugation and plastic adherence as described in our previous studies [29].

### Characterization of immunophenotypic properties of MenSCs *versus* BMSCs

The expression of CD106, CD166, CD105 and CD146 as mesenchymal and OCT-4 as embryonic stem cell markers and CD45, CD133 and CD14 as hematopoietic cell markers were evaluated by flow cytometric analysis. Briefly, aliquots of 10^5^ cells/100 µl were incubated separately with PE-conjugated mouse anti-human CD133 (clone TMP4; eBioscience, CA, USA), CD14 (clone M5E2; BD Pharmingen, CA, USA), CD106 (clone STA; eBioscience), CD105 (clone 43A3; BioLegend, CA, USA), CD146 (clone P1H12; BD Pharmingen), CD45 (clone HI30; BD Pharmingen) or CD166 (clone 3A6; MBL International, Woburn, MA) for 40 minutes (min). To assess OCT-4 expression, the 0.1% saponin-permeabilized cells with were treated with rabbit anti-human OCT-4 antibody (Abcam) for 40 min followed by 30 min incubation with FITC-conjugated goat anti-rabbit Ig (Sigma). Next, all cell suspensions were fixed in 1% formaldehyde solution and examined using a flow cytometer (Partec PAS, Münster, Germany) in reference to appropriate isotype controls (IgG2a κ for CD14 and IgG1 for CD105, CD146, CD45, CD106 and CD166).

Indeed, cells were fixed in acetone at −20°C for 5 min and then subjected to immunofluorescent staining for OCT-4, vimentin and GFAP. In brief, the fixed cells were permeabilized with 0.4% triton X-100 for 20 min. After washing step, cells were incubated for 1 h at room temperature (RT) with rabbit anti-human OCT-4 polyclonal antibody (Abcam), mouse anti-human vimentin monoclonal antibody (clone V9, 1∶200; Sigma) or rabbit anti-human GFAP monoclonal antibody (clone name∶EP672Y, 1∶250). As reagent negative control, the cells were treated in parallel with the same concentrations of normal rabbit irrelevant IgG for OCT-4 and GFAP and mouse irrelevant IgG1 for vimentin.

Subsequently, the cells were washed three times with PBS and incubated with FITC-labeled goat anti-rabbit IgG (Sigma) or FITC-labeled sheep anti-mouse IgG (Avicenna Research Institute) at RT for 45 minutes in the dark. Thereafter, cells were incubated with 4′, 6 diamidino-2-phenylindole (DAPI; 1∶1000) (Sigma-Aldrich) for nuclear staining. The cells were visualized and photomicrographed using an epifluorescence microscope (Olympus BX51 microscope, Tokyo, Japan) connected to digital camera (Olympus DP71, Tokyo, Japan).

### Multi-lineage differentiation potential of MenSCs and BMSCs

To further characterization of isolated MenSCs in comparison with BMSCs, we assessed differentiation ability of these cells into osteoblasts, chondrocytes and adipocytes as described previously (27, 28). The differentiated cells into osteoblasts were identified by specific histochemical staining for calcium with Alizarin red staining (Sigma-Aldrich). Chondrogenesis was assessed by immunofluorescence staining using primary monoclonal mouse anti-human Collagen type II (clone 5B2.5, 1∶500; Abcam) and FITC-labeled goat anti-mouse IgG (Abcam). Adipogenic-induced cells were stained for fat vacuoles using the Oil red O staining. Control cultures without the differentiation stimuli were maintained in parallel to the differentiation experiments and stained in the same manner.

### Multiplex Ligation-dependent Probe Amplification (MLPA)

To investigate chromosomal stability of MenSCs during passages, MLPA analysis was performed on genomic DNA of cells at passages 2 and 12 using the SALSA MLPA kit P036-E1 Human telomer3 (MRC-Holland, Netherlands) according to the manufacturer's protocol. Briefly, a total of 100 ng of genomic DNA in a final volume of 5 µl was denatured and hybridized to SALSA probe mix, followed by incubation at 60°C for 18 hr. Subsequently, the annealed probes were ligated using provided Ligase-65 mix at 54°C for 15 min. In the next step, 10 µl of ligated products, as template, were used for DNA amplification. The PCR amplicons were run on a Genetic Analyzer 3130 (Applied Biosystems, USA), and the results were analyzed by GeneMarker software version 2.4 (SoftGenetics, USA). The normal pattern was expected to produce a normalized signal value ratio of 1∶1; any value out of the ranges <0.75 or >1.35 was considered as abnormal and corresponded to a deletion or duplication, respectively. In each MLPA reaction, whole blood of adult people with no evidence of genetic anomalies, cancerous tissue of colorectal cancer with chromosomal abnormality and aborted fetus with monosomy 21 were simultaneously used as controls. In addition, all control samples were screened for chromosomal abnormality through karyotyping and a normal 550 GTG banding protocol [30].

### Examination of MenSCs immunogenicity and tumorigenicity

Testing of *in vivo* immune response to MenSCs was carried out in five immunocompetent male C57BL/6 mice aged 6 to 8 weeks. Moreover, five nude mice were used to evaluate MenSCs tumorigenicity. To this end, MenSCs at passages 2–4 were harvested using trypsin-EDTA and cell density was adjusted to 1×10^6^ cells/ml in serum- free medium. After that, 0.2 ml of cell suspension was subcutaneously injected in the dorsolateral flank of C57BL/6 mice and nude mice. After two weeks, 20–30 µl of sera was obtained from C57BL/6 mice and sera immunoreactivity to cultured MenSCs was evaluated using immunofluorescence staining.

In order to test tumorigenicity potential, the treated nude mice were scarified and autopsied 12 weeks after cells injection. The inoculation site from the deep aspect was inspected and excised. Moreover, to evaluate the metastatic potential of the MenSCs, the ipsilateral axillary lymph node, spleen, liver, lung, kidneys and heart were excised for histological examination using hematoxylin and eosin (H & E) staining. All procedures were approved by the animal care and ethics committee of Avicenna Research Institute.

### Hepatogenic differentiation

**Figure 1:**
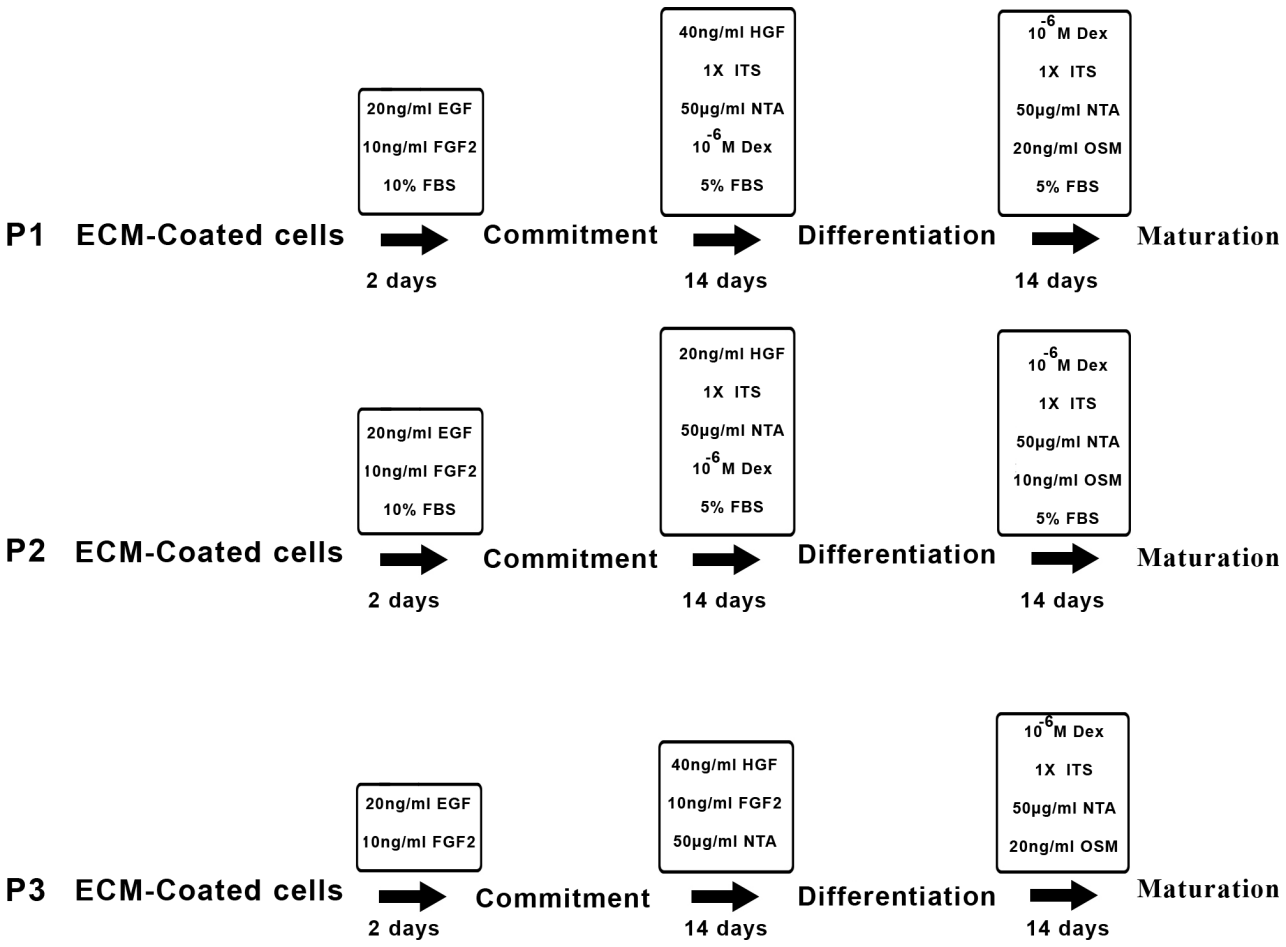
Schematic diagram of three-stage differentiation protocols. Both MenSCs and BMSCs were sequentially treated by three combinations of cytokines, growth factors, and hormones under commitment, differentiation and maturation steps.

MenSCs and BMSCs were plated at a density of 1.5×10^4^ cells/cm^2^ in 1% ECM gel (derived from Engelbreth-Holm- Swarm mouse sarcoma, Sigma-Aldrich)-coated T-25 flasks. In order to induce hepatogenic differentiation, sequential exposures to three different combinations of cytokines, growth factors and hormones were examined. In the commitment step of protocol 1 (P1), 60–70% confluent MenSCs cultured in DMEM-F12 medium were supplemented with 10% fetal bovine serum (FBS) and fortified with 20 ng/ml EGF and 10 ng/ml b-FGF (Sigma-Aldrich) for 2 days prior to differentiation step. Differentiation was induced by treating cells with 5% FBS-supplemented media containing 10^−7^ M Dexa, 1% (insulin, transferrin, selenium pre-mix) ITS+1, 50 µg/ml NTA and 40 ng/ml HGF (all from Sigma- Aldrich). After 14 days, differentiation step was followed by maturation step using a substituted media containing 5% FBS, 10^−7^ M Dexa, 1% ITS +1 and 20 ng/ml OSM (Sigma- Aldrich) for an additional 14 days. Protocol 2 (P2) was done in a similar manner as P1, but with half concentration of HGF (20 ng/ml) and OSM (10 ng/ml). In protocol 3 (P3), cells at 100% confluence were serum-deprived during the treatment period. In this protocol, cells were committed in serum-free DMEM medium supplemented with 20 ng/ml EGF and 10 ng/ml b-FGF for 2 days. Differentiation and maturation steps were conducted as in P1, but in serum-free media. Media in all culture conditions were changed every two days and each protocol lasted up to 30 days. Control cultures without the differentiation stimuli were maintained in parallel to the differentiation experiments in the same manner. The isolated hepatocytes from aborted fetuses (8–12 weeks) or HepG2 cell line were used as positive control. The summary of the differentiation protocols is shown in Figure 1.

### Immunofluorescence staining of albumin (ALB), cytokeratin 18 (CK-18) and Alpha-fetoprotein (AFP)

On the day 30 of differentiation, cells were harvested enzymatically, using 0.25% trypsin-EDTA solution. Cytospins were prepared by centrifugation of the cell suspension onto poly-L-lysine coated glass slides. The cells (10^4^ cells per slide) were fixed in acetone at −20°C for 5 min and incubated overnight at 4°C with primary monoclonal mouse anti-human ALB (clone HAS-11, 1∶100; Sigma-Aldrich), AFP (clone C3, 1∶200; Sigma-Aldrich) or CK-18 (clone RGE53, 1∶100; Chemicon International, Temecula, CA) antibodies. As negative control, cells were treated in parallel with the mouse irrelevant IgG2a for ALB and AFP and IgG1 for CK-18 at the same dilutions. Subsequently, the cells were washed three times with PBS and incubated with FITC-labeled sheep anti-mouse IgG (Avicenna Research Institute) at RT for 45 min in the dark. After washing with PBS, the cells were processed as for OCT-4 immunostaining. Human HepG2 hepatoma cells (National Cell Bank, Pasteur Institute, Tehran, Iran) were simultaneously stained for ALB and CK-18 and considered as positive control. The green fluorescence intensity of 30 protein marker/DAPI positive cells was determined using ImageJ software (ImageJ, NIH, Bethesda, Maryland, USA, http://imagej.nih.gov/ij/, 1997–2012).

### Quantitative real-time reverse transcription–polymerase chain reaction (QRT-PCR)

Expression patterns of mature hepatic markers, *ALB*, tyrosine aminotransferase (*TAT*), cytokeratin-18 (*CK-18*) [2], [18], [23], [31], [32] and cytokeratin-19 (*CK-19*) as oval cell marker [33], [34], [35] were investigated in MenSCs and BMSCs, differentiated under different protocols in reference to untreated cells, using qRT-PCR. Isolated hepatocytes from human aborted fetuses (8–12 weeks) were used as positive control. Total RNA was extracted from 7±2×10^5^ undifferentiated and differentiated cells using RNEasy mini kit (Qiagen, CA, USA) per the manufacturer's instructions. Reverse transcription was performed using 2 µg DNAse (Fermentas Inc, MD, USA)-treated RNA, 1 µl SuperScriptTM II Reverse Transcriptase (200 U) (Life Technologies, CA, USA), 20 pM N6 Random-Hexamer, 20 pM dNTP Mix, 4 µl 5× First Strand buffer, 2 µl Dithiothreitol (0.1 M) and 1 µl RiboLockTM RNase inhibitor (all from Fermentas Inc) in a thermocycler (Eppendorf, Germany) at 25°C for 10 min, 42°C for 50 min and 70°C for 15 min. Next, 2 µl of cDNA was mixed with 1× SYBR Premix EX TaqTM (Takara Bio Inc, Japan), 0.2 µM of each primer [16] and 0.4 µl ROXTM Reference Dye 50×, and amplified using a 7500 Real-Time PCR System (Applied Biosystems, MA, USA) as follows: initial denaturation at 95°C for 10 seconds (sec), 40 cycles of a two-step PCR (95°C for 5 sec, 60°C for 30 sec), dissociation stage at 95°C for 15 sec, 60°C for 1 min and 95°C for 15 sec. The amplified genes were sequenced by 3130 Genetic Analyzer (Applied Biosystems, CA, USA). Mean efficiencies and crossing point values for each gene was determined with LinRegPCR (version 11.0) [36] and normalized to values for GAPDH in differentiated cells in reference to undifferentiated cells using relative expression software tool-2009 (REST-2009) (available at http://www.gene-quantification.info).

To assess expression of *CYP7A1*, 1 µL of cDNA was admixed with 12.5 µL reaction master mix (Amplicon) and 1 µL of each primer (Table 1). After initial denaturation at 94°C for 3 min, PCR amplification was continued at 94°C for 30 s, 60°C for 30 s, and 72°C for 30 s for total cycles of 35, and a final extension was performed at 72°C for 7 min. GAPDH amplification was used as an internal standard. The amplified DNA fragments were electrophoresed on a 2% agarose gel and visualized by an ultraviolet transilluminator (Uvitec- USA). For semi-quantitative determination, specific band density was normalized to that of the corresponding GAPDH using AlphaEase software (Genetic Technologies, Inc.).

**Table 1:** Sequences of the primers used for analysis of cells differentiation into hepatocyte-like cells.

Name of Gene	Sequence	Product size (bp)	NCBI Accession number
*ALB*	F 5′-GAGACCAGAGGTTGATGTGATG-3′	186	NM_00047
	R 5′- AGGCAGGCAGCTTTATCAGCA-3′		
*CK-18*	F 5′-TTGATGACACCAATATCACACGA-3′	202	NM_000224
	R 5′-TATTGGGCCCGGATGTCTG-3′		
*CK-19*	F 5′-GCGGCCAACGGCGAGCTA-3′	154	NM_002276
	R 5′-GCAGGACAATCCTGGAGTTCTC-3′		
*CYP7A1*	F 5′-CAAGCAAACACCATTCCAGCGAC-3′	388	NM_000780
	R 5′-ATAGGATTGCCTTCCAAGCTGAC-3′		
*TAT*	F 5′- 5CTTCTGGGGCTATGTACCTCA-3′	165	NM_000353
	R 5′- GGACTGTGATGACCACTCGGAT-3′		
*GAPDH*	F 5′-CTCTCTGCTCCTCCTGTTCG-3′	114	NM_002046
	R 5′-ACGACCAAATCCGTTGACTC-3′		

ALB: Albumin, CK-18: Cytokeratin-18, CK-19: Cytokeratin-19, CYP7A1: Cytochrome P450 7A1, TAT: Tyrosine aminotransferase, GAPDH: Glyceraldehyde 3-phosphate dehydrogenase.

### Albumin secretion

We quantified ALB in the media to assess ALB secretion ability of differentiated cells, a reliable assay to evaluate hepatic metabolic function [2], [32]. Sample culture media was collected on days 0, 15, 20 and 25 of differentiation and measured by human ALB ELISA quantitative Kit (Immunology Consultant Lab, OR, USA) according to the manufacturer's instructions. The measurements were performed in triplicate.

### Glycogen storage

Intracellular glycogen was analyzed by Periodic Acid-Schiff (PAS) staining. The cells were seeded on poly L-lysine mounted slides and fixed in 4% paraformaldehyde. The slides were oxidized in 1% periodic acid for 5 min and rinsed three times in deionized water. Slides were then treated with Schiff's reagent (Sigma-Aldrich) for 15 min and subsequently counterstained with Mayer's hematoxylin for 1 min. The slides were inspected carefully by three independent persons under a light microscope (Olympus BX51) and scored according to intensity level of PAS stain from 1+ to 4+.

### Statistical analysis

All experiments were performed using cells at passages 2–4 from 3–6 donors. All measurements were performed in triplicate. The results of flow cytometry were presented as median ± range. ALB quantification using ELISA was analyzed using Mann-Whitney *U*-test and the data were expressed as mean ± standard deviation (SD). For the statistical analysis, the SPSS 13 software was used and *P* value<0.05 was considered significant.

Statistical analysis of relative gene expression results in real time PCR was performed using REST© freeware according to formula presented by Pfaffl et al [37].

## Results

### *In vitro* and *in vivo* characterization of cultured cells

**Figure 2:**
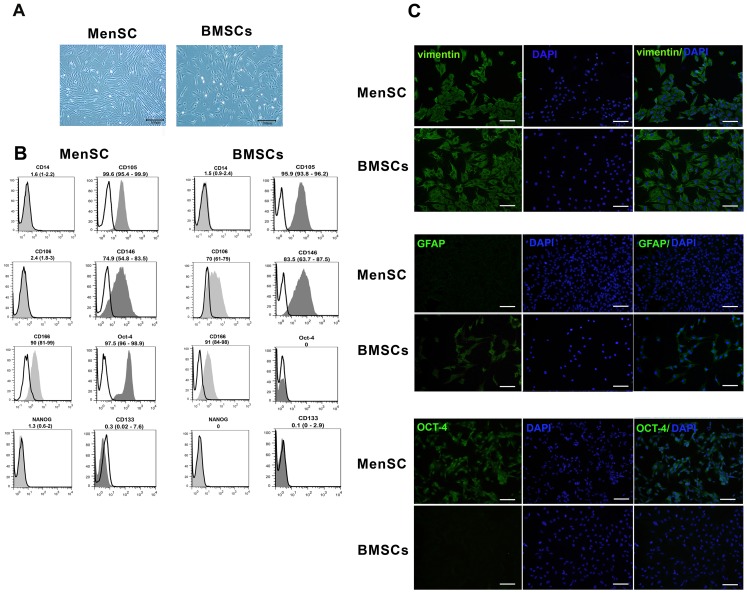
Phenotypic characterization of the MenSCs and BMSCs. (A) Morphological appearance of cultured MenSCs and BMSCs at passage 1; Scale bar: 100 µm. (B) Representative histograms of MenSC and BMSCs immunophenotyping by flow cytometry. CD markers are demonstrated in gray and the respective isotype control is shown as colorless. The results are presented as median (range) of 3–5 independent experiments. (C) Immunofluorescence staining of OCT-4, vimentin and GFAP in cultured MenSCs and BMSCs. Scale bar: 100 µm.

The MenSCs like BMSCs had a same spindle-shaped and fibroblast-like morphology (Fig. 2A). The immunophenotyping analysis of the cells by flow cytometry and immunofluorescence staining revealed that MenSCs in a similar manner with BMSCs typically expressed CD105, CD166, CD146 and vimentin, while unlike BMSCs failed to express CD106 and GFAP. In contrast, expression of OCT-4 was only observed in MenSCs. Lack of CD133 and CD45 in cultured cells reflected a non- hematopoietic stem cell phenotype (Fig. 2B and 2C).

**Figure 3:**
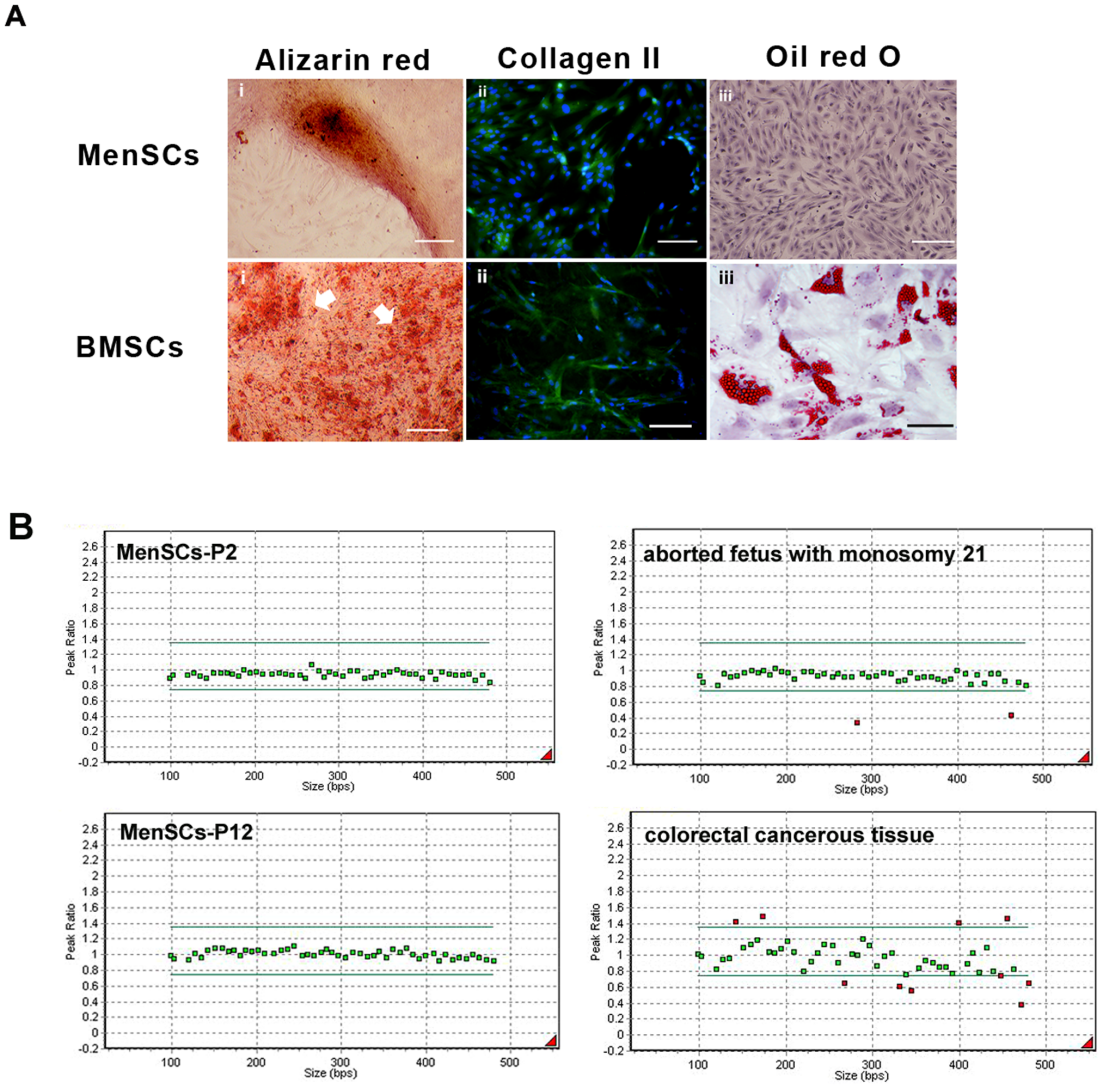
Evaluation of multipotency and chromosomal stability of isolated MenSCs *versus* BMSCs. (A) MenSC and BMSCs differentiation into osteoblasts (ii), chondrocytes (iii) and adipocytes (iiii) judged by Alizarin red staining, immuostaining of Collagen type II and Oil red O staining, respectively; Scale bar: 100 µm. (B) Chromatograms illustrating no chromosomal aberrations in MenSCs at passage 12 compared to cells at passage 2. GeneMarker plots showing results of MLPA analysis. Green lines illustrated the upper and lower limits of acceptable ranges of variations in MLPA analysis. Green dots show the chromosomal locations which are balanced and the red dots in the upper side of the plots show chromosomal gain and red dots in lower side of the plots show chromosomal loss.

MenSCs differentiated into chondrocytes had strong immunoreactivity with monoclonal antibody against Collagen type II in a similar manner with differentiated BMSCs. Mineralization was also pronounced in both osteoblast-differentiated MenSCs and BMSCs as showed by Alizarin red staining. However, the mineralization potential of MenSCs was much lower than that of BMSCs. Meanwhile, unlike formation of oil droplets in differentiated BMSCs, Oil red O staining of the differentiated MenSCs into adipocytes was negative (Fig. 3A).

MLPA analysis showed that MenSCs, in contrast to positive controls, maintained diploid phenotype without chromosomal aberrations during passages (Fig. 3C).

**Figure 4:**
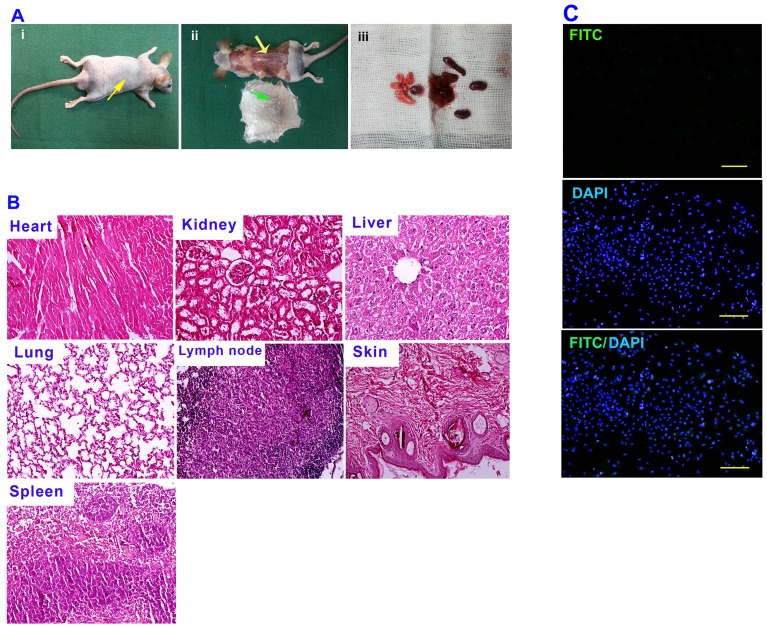
*In vivo* assay of tumorigenicity and immunological reaction of MenSCs. (A) 2×10^6^ cells were subcutaneously injected in the dorsolateral part of the flank of nude mice (i), No tumor formation was observed in treated mice (ii), The excised tissues were fixed in buffered formalin, embedded in paraffin, and sectioned in 5-µm sections (iii). (B) The sectioned tissues were evaluated using H & E staining. (C) Immunoreactivity of mice sera to cultured MenSCs was evaluated using immunofluorescence staining. The cells (2×10^4^ cells per slide) were fixed in acetone at −20°C for 5 min and were incubated for 1 hour at 4°C with mice sera. Subsequently, the cells were washed three times with PBS and incubated with FITC-labeled sheep anti-mouse IgG at RT for 45 min in the dark. DAPI was used for nuclear staining.

According to histological examination of nude mice injected with MenSCs (Fig. 4A & B), no evidence of tumor growth was found in inoculation site and the examined tissues had no morphological characteristics of tumor as judged by H & E staining. Moreover, analysis of immunoreactivity of mice sera to cultured MenSCs revealed no positive reaction in immunofluorescence staining (Fig. 4C).

### Phenotypic characterization of differentiated cells into hepatocyte-like cells

**Figure 5:**
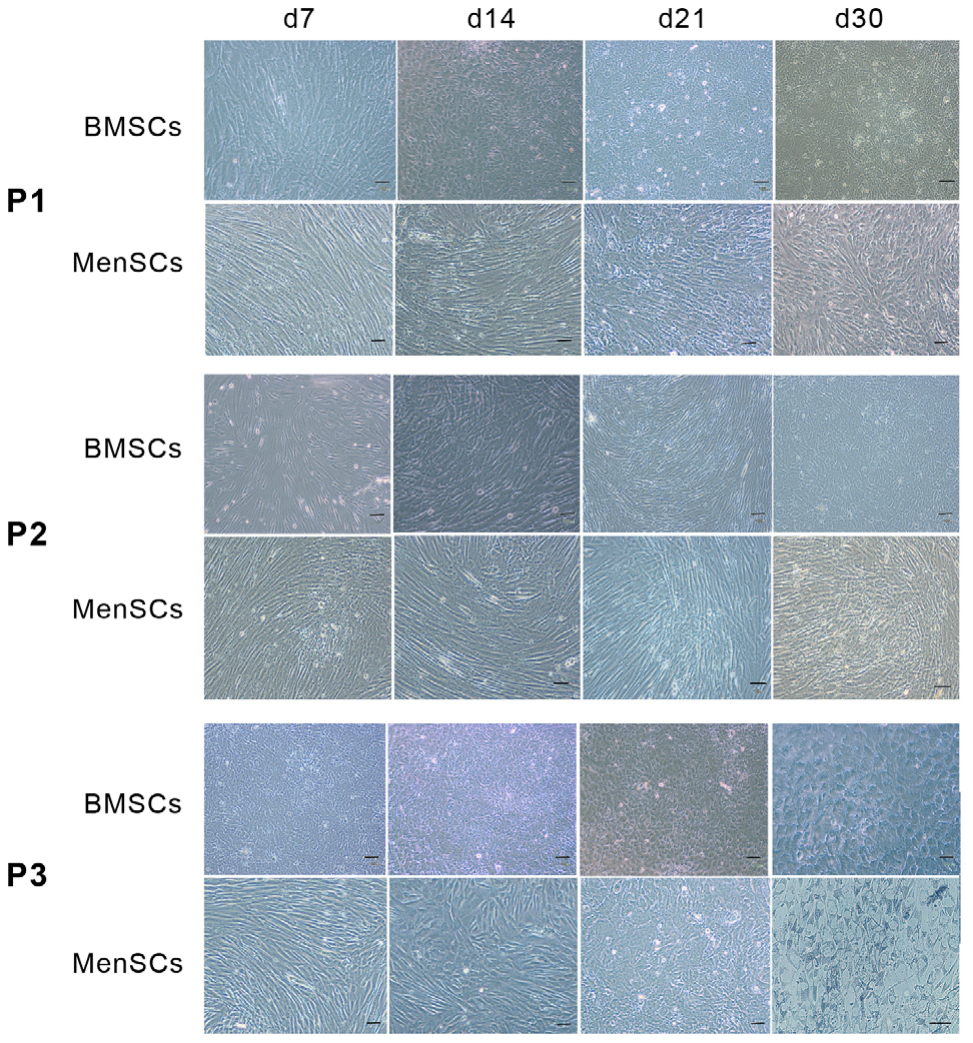
Morphology of MenSCs compared to BMSCs during differentiation by three protocols. The gradual change of MenSC morphology compared with BMSCs under each differentiation protocol (P1–P3) has been shown by phase contrast photographs. Scale bar: 100 µm.

Morphological assessment of the cells using phase contrast microscopy (Fig. 5) showed that under all differentiation conditions, the fibroblast-like morphology of MenSCs and BMSCs did not change significantly during the commitment step. The hepatocyte-like morphology, evidenced by binucleation and cytoplasmic granulation, was observed when both cell types were exposed to differentiation media under protocols 1 and 3 (P1 and P3), with more polyhedral contours and binuclation under P3 compared to P1. These morphological changes were more obvious in differentiated MenSCs compared to differentiated BMSCs. The MenSCs and BMSCs cultured under P2 did not exhibit significant changes in cell morphology during differentiation.

### Immunofluorescent staining of Albumin, CK18 and AFP

**Figure 6:**
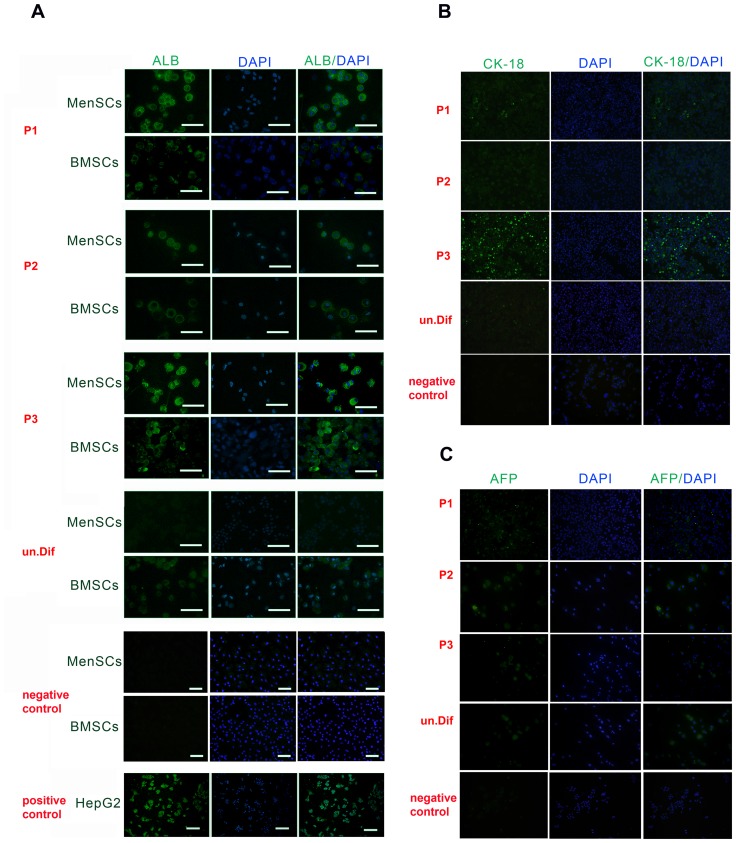
Immunofluorescent staining of differentiated MenSCs and BMSCs. Expression of ALB in differentiated MenSCs and BMSCs (A) and expression of CK-18 (B) and AFP (C) in differentiated MenSCs was examined by immunofluorescence staining with DAPI nuclear staining; scale bar: 100 µm. As negative control, cells were treated in parallel with the mouse irrelevant IgG2a for ALB and AFP and IgG1 for CK-18. Human HepG2 hepatoma cells were considered as positive control.

Albumin (ALB) was not detectable in undifferentiated MenSCs or BMSCs, whereas there was a significant level of ALB expression at the end of differentiation process in both MenSCs and BMSCs under all conditions. The intensity of ALB expression in MenSCs was dependent on differentiation protocol, with the highest level under P3 (44.86±18.34) and lowest under P2 (18.85±4.15). The mean intensity of ALB expression was 27.45±10 in differentiated cells under P1 (Fig. 6A). Nonetheless, the differentiated MenSCs showed a considerably higher level of ALB expression compared to differentiated BMSCs under P1 and P3. The mean intensity of ALB expression was highest when BMSCs differentiated under P3 (25.26±15.2) and lowest (12.85±3.2) in differentiated BMSCs under P2.

Expression of CK-18 in differentiated MenSCs under P1 and P2 was not different with undifferentiated cells, but, the treated MenSCs using P3 showed significantly higher expression level of this protein compared with other groups (Fig. 6B). In contrast, relatively the same levels of AFP expression were observed in MenSCs differentiated by either of protocols and undifferentiated cells (Fig. 6C).

### Hepatic gene expression

**Figure 7:**
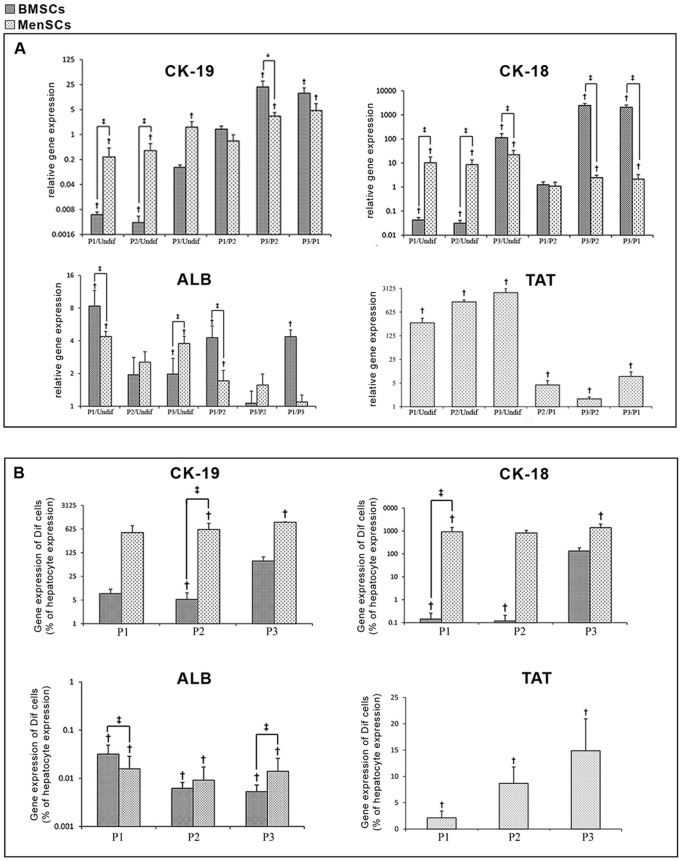
Quantitative RT-PCR results of differentiated cells using three protocols. (A) Data of differentiated MenSCs and BMSCs were normalized to corresponding GAPDH and calculated in reference to undifferentiated cells. Each bar in each differentiation protocol represents the gene expression in ratio to undifferentiated cells (the first three pairs of bars). The second three pairs of bars represent comparisons of a given marker expression between two protocols in each stem cell. † indicates significant difference between differentiated and undifferentiated status of the same stem cell (*P*<0.05), ‡ indicates significant difference (*P*<0.05) between MenSCs and BMSCs. (B) Relative gene expression of differentiated cells compared to isolated adult hepatocytes. Results are shown as % of hepatocyte expression level.

As shown in figure 7A, the MenSCs showed a significant up-regulation of *ALB* gene following differentiation under P1 (4.37±0.50 fold, *P* = 0.001) and P3 (3.7±0.63 fold, *P* = 0.001), whereas no significant change was detected in *ALB* expression induced by P2 compared to undifferentiated MenSCs (2.54±0.6, *P* = 0.17). While the up-regulation level of *ALB* gene in differentiated MenSCs by P1 was ∼2 fold less than that in differentiated BMSCs, up-regulation level of this gene was approximately 3 fold higher in differentiated MenSCs under P3 compared to differentiated BMSCs (*P* = 0.001). In addition, a significant up-regulation of *CK-18* mRNA was observed in both MenSCs and BMSCs differentiated under all protocols (*P* = 0.001). The up-regulation level of this gene in differentiated MenSCs under P1 and P2 was respectively ∼10 and ∼8.6 fold higher compared to differentiated BMSCs (*P* = 0.005). Although, the up-regulation level of *CK-18* mRNA in both differentiated MenSCs and BMSCs by P3 was significantly greater than that of cells differentiated under P1 or P2 (*P* = 0.001), differentiated MenSCs under P3 exhibited significantly lower level of CK-18 in comparison with treated BMSCs under the same protocol (*P* = 0.001). Moreover, a slight up-regulation of CK-19 was observed in differentiated MenSCs under all protocols (P1: 0.23±0.18; *P* = 0.001, P2: 0.35±0.2; *P* = 0.001 and P3: 1.6±0.69; *P* = 0.001). The up-regulation level of CK-19 in treated MenSCs under P1 and P2 was further than differentiated BMSCs (*P* = 0.001). Furthermore, a typical up-regulation of TAT gene was beheld in the all diverted MenSCs in ratio to undifferentiated cells. The TAT over-expression was higher in cells differentiated under P2 (1251±170, *P* = 0.001) and P3 (2331±777, *P* = 0.005) compared to the cells induced by P1 (300±108, *P* = 0.001).

Although the expression level of *ALB* gene in both differentiated BMSCs and MenSCs under all protocols was lower than those observed in isolated fetal hepatocytes, the expression levels of *TAT*, *CK-18* and *CK-1*9 genes in both differentiated cells were significantly higher than those of isolated fetal hepatocytes in a protocol-dependent manner (Fig. 7B).

### Functionality assay

**Figure 8:**
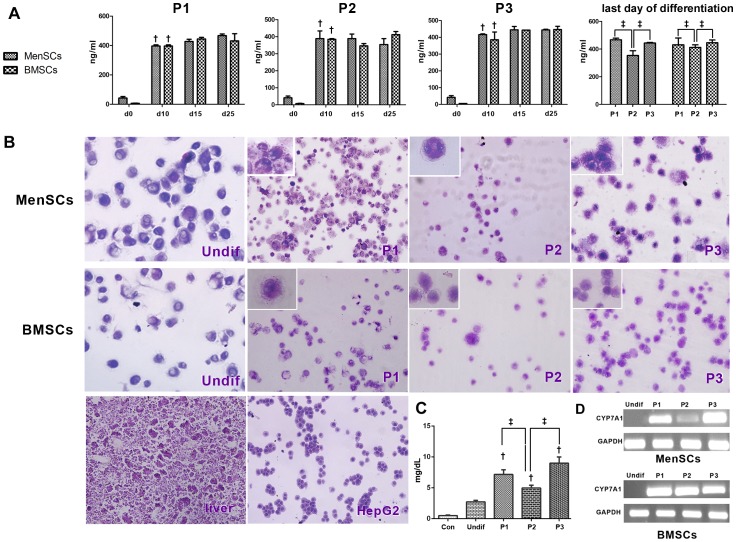
Functionality characteristics of differentiated MenSCs compared to BMSCs. (A) ALB levels (ng/ml/48 h) in cell supernatants at days 0, 10, 15, and 25 of differentiation. † indicates significant difference between specified day and the previous time period of differentiation in the same stem cell (*P<0.05*), ‡ indicates significant difference (*P<0.05*) between differentiated MenSCs and BMSCs at last day of differentiation. (B) PAS staining of glycogen storage in fetus liver and HepG2 as positive control, undifferentiated and differentiated MenSCs and BMSCs by various protocols (P1–P3). (C) Expression pattern of Cytochrome P450 7A1 (*CYP7A1*) in reference to GAPDH in differentiated MenSCs and BMSCs by various protocols. Undif: undifferentiated cells, W: water.

Glycogen storage and ALB secretion were evaluated to determine whether the hepatocyte-like cells also had functional features related to hepatocytes. Our analysis for ALB secretion indicated that undifferentiated MenSCs and BMSCs did not secrete ALB, whereas after hepatic induction under all protocols, a time-dependent increase in the level of secreted ALB was observed (Fig. 8A). Although ALB level had no significant difference between differentiated MenSCs and BMSCs, there was a significant difference in ALB level of differentiated cells depending on the differentiation protocol. In both cell types, the secreted ALB by differentiated cells under P1 (MenSCs: 467.3±16, BMSCs: 431±13.86) and P3 (MenSCs: 443.95±6.15, BMSCs: 446.25±5.47) was in a higher level compared to cells induced with P2 (MenSCs: 350±28.7, BMSCs: 398±5.23)

Based on the findings of PAS staining (Fig. 8B), all differentiation protocols resulted in glycogen accumulation in derived MenSCs or BMSCs, however the intensity of PAS staining varied dependent on the differentiation protocol and cell type. The highest PAS intensity was in both cell types was observed in P3 (4+). While the intensity of PAS staining had no gross difference between BMSCs induced by P1 and P2 (both 2+), the differentiated MenSCs under P1 exhibited higher level of glycogen deposits compared to those induced by P2 (3+ versus 1+). Furthermore, mRNA expression of Cytochrome P450 7A1 (*CYP7A1*) was examined for further functional characterization of differentiated hepatocyte-like cells. As shown in figure 8C, MenSCs in undifferentiated state did not express the *CYP7A1* gene, however, expression of this marker was detectable on the last day of differentiation process under all protocols. Unlike differentiated BMSCs that expressed high levels of *CYP7A1* under all protocols, differentiated MenSCs exhibited a protocol-dependent variation in *CYP7A1* expression. Interestingly, in consistence with data obtained by other functionality tests, differentiated MenSCs under P3 expressed the highest level of *CYP7A1* gene.

## Discussion

Menstrual blood is an interesting stem cell source in the field of regenerative medicine and tissue engineering, because it is abundant and easy to access. Furthermore, MenSCs isolated from MB can be expanded in quantities relevant to clinical applications without chromosomal abnormalities. Non-immunogenic nature and lack of tumor formation capability, as shown in our study, are among other outstanding advantages that make MenSCs a suitable candidate for cell therapy. While current reports of animal studies on MenSCs-based therapy for some diseases such as cardiac [38], muscular [39] and nervous system disorders [40], [41] hold promise for cell-based therapy using these stem cells, there is very limited information about MenSCs potential to generate hepatocytes [8], [16], particularly compared with other known stem cells such as BMSCs. In this study, some protocol-dependent differences were beheld between hepatogenic differentiation potential of MenSCs and BMSCs.

We showed recently that MenSCs possess some markers of mesenchymal stem cells such as CD44, CD73, CD29 and CD90 but fail to express STRO-1 [27], [28]. To further characterization of these cells, in this study we evaluated expression of other mesenchymal and embryonic stem cell markers. The high expression level of BMSCs markers such as CD146, CD166, CD105 and vimentin in parallel to OCT-4, the later being an embryonic stem cell marker, suggests a dual characteristic for MenSCs. On the other hand, lack of some other mesenchymal (STRO-1, CD106, and GFAP) and embryonic (NANOG and SSEA-4) stem cell markers distinguishes MenSCs from these stem cell types [27], [28].

Our preliminary evaluation indicated a trans-differentiation ability of MenSC population into chondrogenic and osteogenic lineages in a similar manner with BMSCs. However, differentiation capability of MenSCs to adipocytes was significantly lower than that of BMSCs. Considering aforesaid differences, comparative evaluation of MenSCs and BMSCs in terms of differentiation into other lineages including hepatic lineage would help us to understand other potential distinctive characteristics that they may present. To our best knowledge, such data has not been reported so far. In the present study, to determine hepatogenic differentiation potential of MenSCs especially compared with BMSCs, we developed three protocols (P1-P3) using HGF and OSM, in two combinations with other components in both serum-supplemented and serum-free culture media. All differentiation processes were entirely examined on ECM-seeded cells, considering the well-known growth and hepatogenic differentiation promoting effect of ECM on stem cells [42], [43].

Here we showed that all induced MenSCs using three protocols acquired some features of functional mature hepatocytes such as mRNA and/or protein expression of ALB, CK-18, TAT, CYP7A1 and also ALB secretion and glycogen accumulation (table 2). Moreover, the differentiated MenSCs showed a slight expression of oval cell (mature hepatocyte precursor) markers, CK-19 and AFP [33], [34], [35], suggesting presence of a small portion of these cells in mature hepatocyte population.

**Table 2:** Comparative analysis of hepatic markers expression in MenSCs and BMSCs using three protocols.

Cell type		Differentiated MenSCs			Differentiated BMSCs		
Differentiation protocol		P1	P2	P3	P1	P2	P3
Immunofluorescent staining	ALB	+++	+	++++	++	+	++
	CK-18	+	+	+++	ND.	ND.	ND.
	AFP	+	+	+	ND.	ND.	ND.
QRT-PCR	*ALB*	++	+	++	++++	+	+
	*CK-18*	++	+	+++	+	+	++++
	*CK-19*	+	+	+	+	+	+++
	*TAT*	+	++	+++	ND.	ND.	ND.
	*CYP7A1*	+++	+	++++	+++	++	++
PAS	Glycogen	+++	+	++++	++	++	++++
ELISA	ALB	++	+	++	++	+	++

The up-regulation levels of hepatic markers compared to undifferentiated cells is scored between 1+ to 4+. ND: not determined.

Based on our results, degree of differentiation was protocol-dependent. In comparison with differentiated MenSCs in inducing media containing 20 ng/ml HGF and 10 ng/ml OSM (P2), cells differentiated under higher concentration of these factors (P1) displayed upper level of ALB at the protein level. In addition, the mean intensity of ALB signal in differentiated MenSCs was higher than differentiated BMSCs under P1 and P3. These differences were also reflected in the results of secreted ALB levels. In consistent with these results, expression level of CYP7A1 and ALB mRNA was significantly higher in differentiated MenSCs under P1 in reference to those developed by P2. Moreover, differentiated hepatocyte-like cells were able to accumulate more glycogen in differentiated cells induced by P1 compared to those by P2.

Therefore, although expression level of CK-18 mRNA had no significant difference between induced MenSCs by P1 and P2, accumulative data showed P1 was more efficient to differentiate MenSCs into hepatocyte-like cells compared to P2.

In a similar manner with MenSCs, differentiated BMSCs under P1 showed a higher level of mature hepatocyte markers including ALB mRNA, CYP7A1 mRNA and produced ALB protein compared to those induced by P2. While some parameters such as CYP7A1 and CK-18 and secreted albumin showed no significant differences between differentiated MenSCs and BMSCs, the expression level of ALB and CK-18 at mRNA and/or protein level was significantly different between these two cell types. The greatest expression of hepatic markers in both MenSCs and BMSCs induced by P1 compared to those differentiated by P2 presents an additional evidence of critical role of HGF and OSM in stem cell differentiation which has also been reported by others [29], [26], [44]. On the other hand, it sounds that the source of stem cells serves a fundamental role on expression pattern of mature hepatic markers. In consistent with the this conclusion, significant differences in hepatic differentiation potential were reported between stem cells derived from various origins including placental-derived stem cells and BMSCs [45].

In sharp contrast to the main role of serum in stem cell expansion, serum-free conditions have been applied on a routine basis for hepatic differentiation of embryonic or adult stem cells [46], [47], [26]. In our knowledge, there are no reports available on hepatogenic fate of stem cells in serum-free media compared with that in serum-fortified media. Surprisingly, we found that omission of FBS during three steps of differentiation process (P3) causes typical improvement in functions assigned to hepatocytes. Some morphological characteristics of the differentiated MenSCs under P3, such as binucleation, granulation of the cytoplasm, and formation of polyhedral contours, resembled native hepatocytes. In addition to the morphological appearance, the expression of ALB protein, TAT, CK-18 and CYP7A1 in mRNA and/or protein levels under P3 was typically superior compared to those of MenSCs differentiated under both P1 and P2. Although ALB mRNA and secreted level of ALB showed no significant differences between MenSCs treated under P3 and P1, the further up-regulation of other mentioned parameters suggested more efficacy of P3 than P1 and P2 to develop hepatocyte-like cells from MenSCs. In line with this conclusion, P3-induced MenSCs accumulated more glycogen than those primed by P1 or P2. Although molecular mechanisms governing the higher efficiency of supplemented serum-free in comparison with serum-fortified media for MenSC differentiation remain to be determined, data suggest that FBS deprivation from differentiation media may serve as a triggering factor for MenSCs differentiation.

Compared to differentiated MenSCs, driven BMSCs exhibited more variable expression pattern during differentiation under P1 and P3. While up-regulation level of ALB and CYP7A1 was higher in BMSCs induced by P1 compared to P3, the opposite pattern was found in the case of CK-18 expression and glycogen accumulation. Indeed, BMSCs differentiated under P1 and P3 showed the same levels of ALB secretion. Thus, based on these results superiority of P3 over P1 in BMSCs is not as certain as MenSCs and further studies are needed to counsel a robust notion. In addition, with respect to high costs and safety problems with FBS, serum-free composition of P3 would be advantageous over P1 that contains FBS during differentiation of both MenSCs and BMSCs.

Differentiated MenSCs and BMSCs showed upper level of CK-18, CK-19 and TAT and lower level of ALB compared with isolated fetal hepatocytes. Such inconsistency has also been reported for hepatocyte-like cells derived from iPS cells [48], [49]. It indicates that the other supplementary strategies, such as three-dimensional cultures or co-culture may be required to further induce the acquisition of native mature hepatocyte nature.

Taken together, the evidence presented in this study indicates MenSCs are a stem cell population with unique immunophenotype and differentiation potential and no risk of tumor formation and immunological reaction. We proved MenSCs have capability to generate functional hepatocyte-like cells, but with different pattern compared to BMSCs depending on critical growth factor concentration and culture media condition. P3 enriched in vitro production of functional MenSC-derived hepatocyte-like cells more than P1 and especially P2. However, in the case of BMSCs, P1 and P3 exhibited rather same efficacy. Differences in protocol-dependent expression pattern of hepatogenic markers between these cells may be attributed to different immunophenotypic features and signaling machinery.

On the other hand, regarding overall expression pattern of hepatic markers in differentiated MenSCs with BMSCs, it sounds that hepatic differentiation potential of these two stem cells had no significant predominance to each other. Here again, considering the refreshing nature, accessibility and lack of ethical issues, MenSCs are superior over BMSCs. Further studies using *in vivo* models and more functional tests are needed before a firm conclusion on applicability of MenSCs-derived hepatocyte-like cells in the clinic or could be made.
